# Circulating Melanoma-Derived Extracellular Vesicles: Impact on Melanoma Diagnosis, Progression Monitoring, and Treatment Response

**DOI:** 10.3390/ph13120475

**Published:** 2020-12-18

**Authors:** Stephanie M. Bollard, Cristina Casalou, Chia Yin Goh, Desmond J. Tobin, Pamela Kelly, Amanda McCann, Shirley M. Potter

**Affiliations:** 1 UCD School of Medicine, College of Health and Agricultural Sciences, University College Dublin, Belfield, Dublin 4, Ireland; chia.goh@ucdconnect.ie (C.Y.G.); amanda.mccann@ucd.ie (A.M.); shirleypotter@mater.ie (S.M.P.); 2 The Charles Institute for Dermatology, School of Medicine, College of Health and Agricultural Sciences, University College Dublin, Belfield, Dublin 4, Ireland; cristina.casalou@ucd.ie (C.C.); desmond.tobin@ucd.ie (D.J.T.); 3 UCD Conway Institute of Biomolecular and Biomedical Research, University College Dublin, Belfield, Dublin 4, Ireland; 4 Department of Plastic & Reconstructive Surgery, Mater Misericordiae University Hospital, Eccles Street, Dublin 7, Ireland; 5 Mater Melanoma Group, Mater Misericordiae University Hospital, Eccles Street, Dublin 7, Ireland; 6 UCD School of Veterinary Medicine, College of Health and Agricultural Sciences, University College Dublin, Belfield, Dublin 4, Ireland; pamela.kelly@ucd.ie

**Keywords:** melanoma, exosomes, extracellular vesicle, biomarker, comparative oncology

## Abstract

Malignant melanoma, one of the most aggressive human malignancies, is responsible for 80% of skin cancer deaths. Whilst early detection of disease progression or metastasis can improve patient survival, this remains a challenge due to the lack of reliable biomarkers. Importantly, these clinical challenges are not unique to humans, as melanoma affects many other species, including companion animals, such as the dog and horse. Extracellular vesicles (EVs) are tiny nanoparticles involved in cell-to-cell communication. Several protein and genomic EV markers have been described in the literature, as well as a wide variety of methods for isolating EVs from body fluids. As such, they may be valuable biomarkers in cancer and may address some clinical challenges in the management melanoma. This review aimed to explore the translational applications of EVs as biomarkers in melanoma, as well as their role in the clinical setting in humans and animals. A summary of melanoma-specific protein and genomic EV markers is presented, followed by a discussion of the role EVs in monitoring disease progression and treatment response. Finally, herein, we reviewed the advantages and disadvantages of methods utilised to isolate EVs from bodily fluids in melanoma patients (human and animals) and describe some of the challenges that will need to be addressed before EVs can be introduced in the clinical setting.

## 1. Introduction

Malignant melanoma, one of the most aggressive human malignancies, is responsible for 80% of skin cancer-associated deaths [1]. Unlike most other cancers, the incidence of melanoma is increasing in humans [2]. Current prognostic indicators for melanoma are crude, relying predominantly upon the Breslow thickness, or depth of the tumour. A Breslow thickness of <0.8 mm carries a 10-year survival of 98% [3]. However, such thin melanomas still account for 22–29% of melanoma-related deaths [4,5]. This is partly due to the increased incidence of thinner tumours, as well as an increased emphasis on early detection. However, this also highlights the lack of a clear method of stratifying risk in melanoma. Similarly, once the initial staging of melanoma has been completed, assessment of disease progression and monitoring of treatment response rely upon clinical examination or radiological imaging, often diagnosing progression only once well established. Whilst the early detection of disease progression or metastasis can improve patient survival [6], it remains a challenge due to the lack of reliable biomarkers.

Notably, these clinical challenges are not restricted to humans, as melanoma affects many other species [7], including companion animals such as the dog and horse. Spontaneously occurring canine dermal and oral malignant melanoma show striking similarities with human melanomas and represent a valuable translational animal model [7,8,9]. Parallel subtypes of melanoma in humans, dogs, and horses share similar driving mutations, such as NRAS, TP53, and PTEN [10,11]. As in human medicine, prognostic indicators for canine and equine melanoma are limited and consist mostly of histological grading (cellular differentiation, mitotic index/Ki67 score) and tumour size. Whilst a large percentage of these presentations are benign melanocytic tumours, there are no reliable methods to identify those patients who may progress and develop metastases. *Comparative oncology*, the parallel study of spontaneous animal models, offers a significant opportunity for translational research.

Extracellular vesicles (EVs) are small nanoparticles released by almost all cells examined to date. They harbour a variety of macromolecules such as proteins, lipids, metabolites, DNA, RNA, and microRNAs. They are involved in cell-to-cell communication and play a role in regulating physiological processes such as angiogenesis, coagulation, inflammation, and immune responses [12]. They are classified on the basis of their size, biogenesis pathway, cell of origin, and function. Three main subtypes of EVs are described in the literature: exosomes, microvesicles, and apoptotic bodies. Exosomes are produced by way of the endolysosomal pathway and are released as a result of the fusion of multivesicular bodies (MVBs) with the cell membrane. They are traditionally thought of as 30–150 nm in size and are characterised by markers such as CD81, CD63, TSG101, and Alix [13]. In contrast, plasma membrane-derived EVs are larger in size. Microvesicles (or ectosomes, 50–1000 nm) are produced as a result of outward budding of the cell membrane, whereas apoptotic bodies (up to 2000 nm) are the result of apoptotic blebbing of the cell membrane [14]. Traditionally in humans, small EVs have been characterised by size and expression of molecules such as TSG101, CD81, CD9, or CD63, and the absence of markers associated with the endoplasmic reticulum exemplified by calnexin [13]. Similarly, in other species such as dogs, horses, and cats, CD63 [15,16] and CD9 [16,17,18] have also been identified as markers of EVs. This suggests that the utility and clinical applications of EVs may be relevant across species. Whilst there have been efforts to characterise EV subtypes [19] and their specific subpopulations [20,21], there remains a lack of consensus regarding specific markers of EV subtypes. As a result, the International Society for Extracellular Vesicles recommends that descriptions of EVs are based upon the size, density, biochemical composition, or cell of origin rather than inferring a particular biogenesis pathway [13].

**Figure 1:**
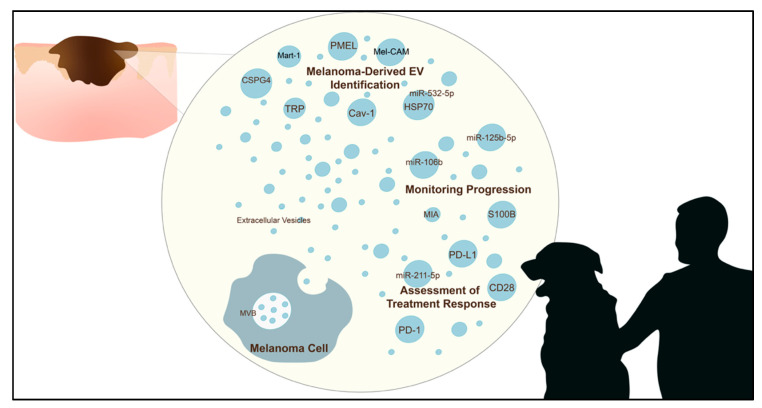
Potential markers of melanoma-derived extracellular vesicles (EVs) that may play a role in diagnosis, monitoring progression, and treatment response in humans and other mammals.

Importantly, EVs play a significant role in disease and are of particular interest in cancer. Their cargo can drive several specialised functions, including those implicated in the control of tumour proliferation, epithelial–mesenchymal transition (EMT), immune evasion, and *pre-metastatic niche* formation in many cancers [22]. In melanoma specifically, it has been demonstrated that EVs play a role in tumour progression [23,24,25,26], and the ability to increase tumour cell migratory capability [27]. This review aimed to explore the translational applications of EVs as biomarkers in melanoma (Figure 1), and their role in the clinical setting in humans and animals. Various combinations of search terms such as “melanoma”, “exosome”, “extracellular vesicle”, “biomarker”, “dog”, “canine”, “horse” and “equine”, were entered into PubMed, Scopus, and Google Scholar and were used to identify articles with a translational application for discussion. As a result, this will not encompass the entire melanoma EV literature but instead includes highlighting markers with potential clinical applications. As the melanoma-associated EV literature in veterinary medicine and animal models is currently limited, the literature discussed herein primarily relates to human melanoma. As much of the literature continues to refer to EVs as “exosomes”, resulting in confusion regarding the type of EV described, this review aimed to predominantly focus on the role of small EVs < 150 nm (exosomes) as a source of potential biomarkers in melanoma. Thus, we refer to them as EVs throughout.

## 2. The Potential Role of EVs as a Source of Biomarkers

Biomarkers are defined as a “characteristic that is objectively measured and evaluated as an indicator of normal biological processes, pathogenic processes, or pharmacologic responses to a therapeutic intervention” [28]. In the setting of cancer, these can be further described as prognostic, predictive, and pharmacodynamic. Prognostic biomarkers allow the prediction of the natural course of cancer, predictive biomarkers may be used to assess whether a patient will respond to a particular treatment, and pharmacodynamic biomarkers can be used to measure the effect of treatment on a tumour [29]. However, for a biomarker to be a viable clinical tool, it must meet a number of criteria. Any cancer biomarker must be reflective of the tumour itself, with a high sensitivity and specificity, whilst its utility should be convenient, minimally invasive, reproducible, and low cost [30].

Extracellular vesicle contents reflect the cell from which they have been derived [31], reproducing both the transcriptome and proteome of the cell of origin [32]. As a result, the number of studies investigating the potential of EVs as biomarkers in disease has increased dramatically over the last 10 years [33]. The proposed tissue specificity of EVs poses an interesting question for researchers. They have been described as a potential “liquid biopsy” [33] due to their detection in multiple different body fluids, in several species, including in plasma, serum, urine, bronchoalveolar lavage, seroma, and milk [13,15,34,35,36,37]. This allows convenient and minimally invasive acquisition.

In cancer, EVs are of specific interest as they are ubiquitous throughout the body and can be viewed as a physiological or pathological bio-print. They can offer a “snapshot” insight into the tumour and metastatic landscape at any given moment. EVs are thought to protect their contents, such as genetic material, from degradation, improving the detection of clinically relevant mutations [38]. Furthermore, in the setting of human melanoma, EVs are intimately involved in regulating the antitumour immune response, angiogenesis, and pre-metastatic niches supportive of metastasis [39]. This appears to be relevant in dogs also. Specifically, Zmigrodzka et al. [40] demonstrated via flow-cytometry that the number of EVs isolated from plasma in dogs with cancer was higher than healthy controls. This study included one dog with melanoma, although it specifically looked at EVs derived from platelets and leucocytes [40]. Whilst the relevance of EVs in melanoma of non-human mammals has yet to be specifically explored, their role in human melanoma progression potentially offers a valuable insight into the tumour biology.

Extracellular vesicles offer significant advantages over other potential liquid biopsy options proposed previously in melanoma. These vesicles are stable when appropriately stored in plasma for at least three months [41]. Currently, many methods of EV isolation exist from plasma or serum [42], and isolation from a single minimally invasive venepuncture has become attainable. As opposed to circulating free DNA (cfDNA) or circulating tumour RNA (ctRNA), which may be as a result of apoptosis or tumour cell injury, the cargo within EVs is thought to be selectively loaded and highly regulated [43]. This may provide further insight into their role in tumour activity. Circulating tumour cells (CTCs), whilst a suggestive feature of epithelial–mesenchymal transition, are challenging to identify, particularly in early stage disease. Whilst circulating melanoma tumour cells can be isolated from blood samples using techniques based upon cell size [44], they are recognised as lacking the cell adhesion molecules often used to identify CTCs [45]. As cutaneous melanoma originates from melanocytes in the skin, principally in the avascular epidermis, access by the tumour cells to the deeper dermal blood vessels is not thought to occur until vertical growth occurs by perforating the basement membrane zone separating the epithelium from the mesenchyme. This is thought to limit access of tumour cells to the circulation during early disease [32], and thus may make CTCs less reliable as biomarkers of melanoma progression in comparison to EVs. In contrast to other components of the tumour circulome, EVs are detectable in early stage disease, with an increase in number associated with increased tumour burden [46,47,48].

## 3. Utility of EVs in Melanoma Diagnosis

In order for melanoma-derived EVs to be a reliable source of biomarkers or a tool in the diagnosis of melanoma, they must first be identifiable within a chosen body fluid. EVs attributed to melanoma have been detected in patient serum, as well as in malignant effusions such as ascites and pleural effusions [49]. The concentration of all EVs identified in the serum of patients with melanoma is higher in comparison to healthy controls [50]. Interestingly, this is the case even when patients show no further clinical evidence of disease postoperatively. Furthermore, it has been hypothesised that the ratio of melanoma EVs to total protein content in plasma could also be used to distinguish between distinct clinical presentations [50]. This suggests that in the setting of melanoma, EVs may be a valuable tool when diagnosing and prognosticating. Moreover, melanoma-derived EVs may better capture primary tumour characteristics in comparison with traditional histological approaches. For example, Cordonnier et al. (2020) demonstrated that while EV-associated programmed death ligand 1 (PD-L1, which binds the PD-1 receptor on activated T, B, and myeloid cells) was identified in the serum of 100% of patients with melanoma (*n* = 30), immunohistochemical evaluation identified PD-L1 in only 67% of tumour biopsies [51]. This suggests that circulating EVs could potentially provide clinicians with a better overview of the dynamic tumour heterogeneity, whilst also protecting the tumour-derived cargo that may otherwise be degraded. 

However, for EVs to be used as reliable biomarkers, it will be essential to detect circulating EVs that are specifically derived from the melanoma tumour. As such, there have been multiple studies that have attempted to define a specific signature melanoma EV, and thus allow their identification in patient serum. Potential clinically relevant markers of melanoma-derived EVs are summarised in Table 1.

### 3.1. Melanoma EV Protein Markers

Several EV-related proteins have been hypothesised to be specific to cancer cells overall, which are also expressed on melanoma-derived EVs [25,32,52,61]. Notably, many cancers may express common proteins in their EVs, as there is evidence that shared tumour antigens can stimulate the immune system across different tumour types and across species [62,63]. A summary of the potentially clinically relevant melanoma-derived EV markers and their functions, as described and identified in this review, is presented in Table 2.

Heat shock protein 70 (HSP70) is a protein involved in protecting the cell from proteotoxic stress. The over-expression of HSP70 is associated with poor prognosis in cancer due to its ability to protect the cell from stress associated with the accumulation of mutant proteins and rapid proliferation [64]. HSP70 has been identified in melanoma-derived EVs [25,32,52] and is also expressed in many different tumour-derived EVs in both humans [65] and dogs [62,66]. In fact, EV-associated HSP70 has been proposed as a potential biomarker for diagnosing and monitoring a variety of solid tumours, such as lung, breast, and ovarian cancer [65], and ongoing clinical trials are utilising a specific peptide aptamer to bind to the extracellular portion of HSP70 and thus capture relevant EVs [67]. If successful, it seems feasible that similar studies could be extended to investigate the role of HSP70 in the diagnosis and monitoring of melanoma. Furthermore, expression of HSP70 in canine malignancies has been reported [68], and its role in canine cancer immunology [69] suggests that this may be extrapolated to other species, including humans. Furthermore, Caveolin-1, a protein involved in mediating the metastasis and progression of cancers in general [61], and melanoma specifically [70], has also been quantified in the plasma-derived EVs of melanoma patients [53] and from melanoma cell lines [27].

Further attempts have been made to define a melanoma-specific EV signature through proteomic studies. However, to date, the majority of these studies have focused on melanoma cell lines [71]. The first of these, performed by Mears et al. in 2004, profiled the EVs of two such cell lines, SK-MEL-28 and MeWo, and demonstrated the presence of melanoma-specific antigens Mel-CAM and Mart-1, as well as the generalised marker HSP70 [52]. Mart-1, in association with tyrosinase-related proteins (TRP), has also been associated with melanoma-derived EVs previously [72]. Furthermore, this combination of MART-1, TRP-1, and TRP-2 have been used as evidence to confirm EV origin from melanoma cells [54].

The large proteomic study of Hurwitz et al. [32], assessing the NCI-60 cell line panel, identified a potential melanoma-specific EV marker protein, namely, premelanosome protein (PMEL), in all nine melanoma cell lines investigated [32]. PMEL (also known as gp100, Silver) is a pigment cell-specific protein, involved in the formation of amyloid-like fibrils in melanosomes, the organelle involved in melanin synthesis [73]. PMEL has since been identified in subsequent proteomic studies of melanoma EVs [74]. PMEL has also been identified in three of seven melanoma cell lines studied by Lazar et al. [27], although interestingly not in EVs isolated from SK-MEL-28 [27] as reported by Hurwitz et al. [32]. This suggests that the presence of these markers may also be related to other experimental (e.g., in vitro) conditions. In order for any of these markers to be relevant, they must also be melanoma-specific, easily identifiable, and clinically useful. PMEL has been identified in the EVs isolated from the ascites of a small number of melanoma patients [49]. Peinado et al. [25] developed a potential melanoma-specific EV signature, stating the importance of TRP-2, VLA-4, HSP70, HSP90, and MET in circulating EVs. This was initially developed using mass spectrometry analyses of the cell culture supernatant of five highly metastatic melanoma cell lines, although subsequently also identified as a marker of advanced melanoma in human patients [25]. Furthermore, Sharma et al. [55] reported utilising the presence of the transmembrane protein CSPG4 to isolate melanoma-specific EVs from patient plasma, using antibody precipitation to target the CSPG4 epitope expressed on melanoma cells [55]. This demonstrated the ability to differentiate the plasma of melanoma patients from healthy donors, as EVs from healthy donors were found to be negative for CSPG4 [50]. Interestingly, CSPG4 has been reported to be a potential biomarker in canine melanoma [75,76], and thus this epitope may also apply to other animal models of melanoma. However, the reported specificity of CSPG4 for melanoma in humans remains in question, as it has also been identified in the EV isolates of other cancer cell line models, such as oral squamous cell and pancreatic adenocarcinoma [74].

### 3.2. Genomic Markers

In an effort to find genomic markers of melanoma EVs, researchers have performed several studies in vitro (Table 3). In comparison to EVs isolated from normal melanocytes (*HEMa-LP*, *NHEMc)*, the EVs of melanoma cell lines have been shown to contain increased mRNA levels for inflammatory chemokines and decreased levels of mRNA for TRP-1 and ATP-binding cassettes (ABCB5) [57]. Interestingly, both of these features are related to melanoma initiation and progression. Similarly, mRNA expression of the major histocompatibility antigen HLA-C expression appears to be downregulated in EVs isolated from melanoma cell lines in comparison to normal melanocytes (*HEMa)*, suggested as an indicator of poor immunogenicity [79].

In addition to mRNA, EVs have also been reported to contain microRNAs (miRNA, miR). These non-coding RNAs, which were initially discovered in *Caenorhabiditis elegans* [80], are involved in the regulation of gene expression. While they have been demonstrated to exist stably in body fluids such as saliva, urine, breast milk, and blood, it is suggested that their packaging within EVs can protect them from degradation [81]. Furthermore, EV-associated miRNAs are altered in other canine diseases such as leishmaniasis [82] and mammary cancer [17,83], again suggesting that the analysis of EV-associated miRNA may be relevant in the monitoring of disease across species. As EV-associated miRNAs reflect the contents of their parent cell, there have been multiple attempts to define cell type -specific miRNA signatures in different malignancies, with the aim of elucidating the role of oncogenic and oncosuppressive EV-derived miRs [84].

The miRNA profiles of melanoma- and melanocyte-derived EVs were compared in vitro by Xiao et al. [57] Utilising miRNA arrays, the authors demonstrated increased levels of 130 miRNAs and decreased levels of 98 miRNAs in EVs derived from the melanoma cell line A375, compared to the EVs derived from the normal adult primary melanocytes cell (HEMa-LP). Many of these dysregulated miRNAs were associated with cellular growth, proliferation, movement, and cell death, and included miR-21, miR-23, miR-let7a/c, miR-138, miR-125b, miR-222, and miR-494 [57].

While it has been reported that miR-125b-5p is the one of most abundantly and significantly enriched miRNAs in three melanoma cell lines (*WM9*, *WM35*, and *WM902B)* in vitro (in comparison to normal melanocytes) [59], others have reported that lower serum levels of EV-associated miR-125b are associated with melanoma in clinical studies [60]. As such, the role of miR-125b in identifying melanoma-related EVs remains unclear. For example, while Alegre et al. (2014) reported that patients with advanced melanoma had lower levels of EV-associated miR-125b compared to healthy controls [60], Pfeffer et al. [56] found no difference in the serum levels of EV-associated miR-125b between patients with melanoma and those without. The latter study also reported higher levels of EV-associated miR-17, miR-19a, miR-21, miR-126, and miR-149 detected in the plasma of individuals with metastatic sporadic melanoma in comparison to healthy controls [56]. Other suggested markers of circulating melanoma-derived EVs in the clinical setting include miR-191 and miR-let-7a [85], and miR-494 [86].

In order to make sense of these data and to identify potential melanoma-specific EV biomarker signatures, researchers have suggested combinations of these genetic and proteomic markers, as well as the incorporation of normalised biological controls. Tengda et al. [58] reported a sensitivity of 92% and specificity of 88% when combining EV-derived miR-532-5p and miR-106b in a panel to differentiate patients with melanoma of varying clinical stages from healthy individuals [58].

## 4. Role of EVs in Monitoring Melanoma Progression

Extracellular vesicles play a key role in promoting the progression and metastases of melanoma [23]. They have been shown to drive tumorigenesis and cell proliferation through the upregulation of the PI3K/AKT pathway [87] and downregulation of cell cycle regulators such as p27 [88]. It is becoming increasingly clear that EVs may have value as prognostic biomarkers in monitoring and predicting melanoma progression. This may potentially alert clinicians to recurrence before it is clinically evident, thereby facilitating timely interventions and thus improving patient outcomes [6].

The quantity and size of EVs in the circulation do not seem to be altered by clinical stage in melanoma [25,51,78], although other alterations in the circulating EV profile are apparent. Patients with advanced disease appear to have a higher concentration of protein per particle, both in plasma [25] and exudative seromas [35]. In vitro studies suggest that the cargo of these EVs contains distinct proteins with activities reflecting the stage of melanoma progression and metastases. Lazare et al. demonstrated that the cargo of EVs isolated from metastatic melanoma cell lines contained proteins strongly associated with regulation of the actin cytoskeleton and cell adhesion. This suggests that the cargo of EVs from melanoma cells contain distinct proteins reflecting the stage of progression and metastases. Similarly, other proteins associated with cell adhesion and migration modulation, such as LAMA1 and LAMB1, have been identified in the cargo of EVs from the metastatic melanoma cell line *H3* [74].

Moreover, the composition of EV cargo appears to change with melanoma clinical stage. In a murine melanoma model, the quantity of CD63-positive EVs appeared to increase in relation to tumour burden [53]. Similarly, S100B and MIA proteins have been found at higher levels within the plasma-derived EVs of patients with advanced melanoma, in comparison to those who are disease-free [77]. Moreover, patients with advanced melanoma have also been shown to have lower levels of miR-125b within EVs isolated from serum, compared to those who are disease-free or healthy controls [60]. Similarly, the EV cargo panels described by Tengda et al. [58] and Peinado et al. [25] appear to differentiate melanoma on the basis of the clinical stage, identifying those with advanced disease. Furthermore, the cargo of melanoma-related EVs appears to be altered on the basis of the volume of nodal metastases, with patients with a higher nodal burden displaying a higher seroma-derived EV content of the RAS/RAF/MAPK pathway-related molecules [35].

The level of EV-associated PD-L1 may also allow differentiation of melanoma patients on the basis of their tumour burden. Whilst EV PD-L1 plasma levels did not correlate with the tumour Breslow thickness, subtype, or age of the patient [51], there was an increased level of EV PD-L1 protein overall in the plasma of patients with melanoma in comparison to healthy human controls [51,78]. EV-associated PD-L1 plasma levels also increased with tumour burden in a murine model and in humans, and was higher in metastatic versus non-metastatic melanoma cell lines [78]. PD-L1 therapies may be applied to canine [89,90] and equine melanoma [91] patients, and by extension to other species.

There have been attempts to utilise the cargo of exosomes to predict eventual melanoma progression and prognosis through the correlation with known prognostic indicators. In patients with a thicker melanoma (i.e., higher Breslow thickness), higher levels of miR-126, miR-149, miR-19a, miR-21 [56], miR532-5p, and miR-106b have been identified in their associated EVs [58]. In addition, the detection of BRAFV600E-mutated DNA in the EVs isolated from exudative seroma of melanoma patients correlated with tumour expression of the BRAF mutation [35]. This finding was also shown to predict eventual progression and was hypothesised to be an indicator of residual disease [35].

Although currently underdeveloped in the melanoma field, an integrin signature expressed on melanoma EVs has been suggested to predict future organ-specific metastases, thus playing a role in the uptake of EVs at pre-metastatic sites. In the setting of breast cancer, the development of lung metastases is associated with EV-related ITGß_4_, whereas in the setting of pancreatic cancer, EV-related ITGα_v_ is associated with the development of liver metastases [92]. Charoenviriyakul et al. [93] demonstrated a role for surface proteins, including integrins, in the pharmacokinetics and biodistribution of melanoma EVs in vitro [93]. Further study is required to investigate the relevance of these and other integrins in the development and prediction of eventual melanoma metastases.

## 5. Role of EVs in Monitoring Treatment Response

Extracellular vesicles may also be used to assess treatment response in melanoma. The first-line treatment for a primary presentation of melanoma is surgical excision in humans, as well as in dogs and horses. The impact of the removal of the primary melanoma on the EV profile has not been thoroughly researched to date. However, there is evidence that the reduction of tumour burden, via surgical resection, is associated with a reduction in the concentration of circulating EVs in humans [94].

There have been multiple studies investigating how EVs may play a role in monitoring the patient’s response to oncological treatment. In vitro studies have suggested that the secretion and shedding of EVs may be increased in response to chemotherapy [95], particularly for those EVs containing HSP70 [65]. Similarly, in vitro and murine studies have demonstrated an increased secretion of miR-211-5p containing EVs as a response to treatment with BRAF inhibition with dabrafenib [96]. This suggests that EVs can be used to monitor tumour stress response or injury. Furthermore, these findings argue well for clinical application in humans, and indeed it has been reported that human patients undergoing treatment with chemotherapy may have lower levels of caveolin-1-related EVs [53].

The impact of treatment on EVs in canine and equine melanoma patients has not yet been assessed. Canine and equine patients with a high risk of metastases may be treated by platinum-based chemotherapeutic agents, although often with limited response [97,98]. This is partly due to the chemoresistant nature of melanoma cells, parallel to that seen in humans. However, a high serum EV concentration has been shown to be predictive of response to the CHOP-based (cyclophosphamide, doxorubicin, vincristine, and prednisone) chemotherapy protocol in canine lymphoma patients [99]. It stands to reason that the EV profile in both dogs and horses may also be altered following treatments used in melanoma.

Melanoma is known as a variably immunogenic tumour, with melanoma-derived EVs playing a significant role in modulating the immune response to the tumour, including by influencing the tumour-associated macrophage phenotype [95,100]. Moreover, melanoma-derived EVs containing Tumour necrosis factor-related apoptosis-inducing ligand (TRAIL) or Fas 9 can influence the proliferation and apoptosis of CD8+ T cells, further modulating the patient’s response to melanoma [50]. However, perhaps the most significant advance in the treatment of melanoma has been the advent of immune checkpoint inhibitors, for example, the class of PD-1 inhibitors and CTLA-4 (CD152) inhibitor/antagonists, which have had a very significant impact on melanoma survival [101,102]. Similarly, inhibition of the PD-1/PD-L1 axis in dogs with melanoma has been reported as a potential treatment, with pilot studies demonstrating antitumour response [90].

EV-related PD-L1, which has the same membrane topology as tumour cell PD-L1, has been detected at a higher concentration in the serum of patients who later fail to respond to these immune checkpoint inhibitors [78]. This suggests that melanoma-derived EVs play a role in the peripheral inhibition of the immune system, as it appears that upregulation of PD-L1 may allow cancers to evade the host immune system. Those melanoma patients who do respond to these drugs also show a peak in EV-related PD-L1 levels 3–6 weeks after commencing PD-1 inhibition therapy [78]. Furthermore, circulating levels of EV-related PD-L1 protein and EV-related PD-L1 mRNA both decrease concurrently with radiological evidence of tumour response, and increase with evidence of melanoma progression [51,103]. As such, EV-related PD-L1 measurements may facilitate both prediction and monitoring of response to PD-L1 inhibitors (e.g., by durvalumab, pembrolizumab, atezolizumab, and avelumab). EV-related PD-1 has also been isolated from patient serum, specifically from T cell-derived EVs, isolated using immunoaffinity-based techniques. Specifically, Tucci et al. (2018) demonstrated that a higher basal level of EV-related PD-1 and CD28 from T cells identified patients most likely to respond to inhibition of CTLA-4 (a protein that stops the immune system from attacking cancer cells) by the drug ipilimumab. Patients with a higher basal level of EV-related PD-1 and CD28 from T cells were associated with a higher progression-free and overall survival [104]. Similarly, treatment-associated increase in the level of CD80 on dendritic cell-derived EVs facilitated prognostication and was also associated with a longer progression-free survival, suggesting reactivation of the antitumour immune response [104].

In addition to monitoring treatment responses by measuring levels of EV-related PD-L1 and PD-1, preclinical melanoma studies have suggested that circulating EVs can act as mediators of cellular resistance to chemotherapeutic agents. For example, Federici et al. [105] reported that cisplatin uptake by melanoma cells in vitro was reduced under the acidic extracellular pH conditions that favour both increased EV release and incorporation of cisplatin within melanoma EVs (rather than the melanoma cell itself) [105]. In this way, EV-related pathways appear to be involved in the elimination of and resistance to the anticancer agent cisplatin. Furthermore, it was demonstrated in a murine model that the use of a proton pump inhibitor to reduce acidity can act as a chemosensitising agent, i.e., by reducing the cisplatin content in circulating EVs [105]. Similarly, Cesi et al. [106] demonstrated that resistance to the BRAF inhibitor vemurafenib was transferred via EVs between melanoma cells in vitro through the transport and uptake of a truncated version of the tyrosine receptor ALK [106].

## 6. Methods of Isolation and Identification of EVs

There have been several different methods described for the isolation and characterisation of EVs, resulting in the publication of the Minimal Information for Studies of Extracellular Vesicles Guidelines in 2018 [13]. Given that EVs are released from every cell type studied to date, reliable cancer-specific methods to isolate those EVs are required, and thus several commercial and branded assays have been developed.

The yield of EVs and their composition varies depending upon the methodology, pre-collection treatment, and body fluid chosen. Given the ubiquity of EVs for all our body cells, it is important to note also that most circulating EVs in cancer patients will not be related to their cancer. However, the true proportion of EVs in the circulation that is attributable to the tumour is not known. Serum is deemed to have a higher proportion of platelet-derived EVs in comparison to plasma [107]. Beyond this, even the choice of anticoagulant used for plasma preparation may alter the EV populations. For example, citrate use may result in a higher concentration of platelet-derived EVs [107], but a lower yield of EVs overall [108].

It has also been shown that the method of EV isolation from serum and plasma significantly impacts yield and protein composition [42]. Ultracentrifugation (UC) is one of the most commonly used methods of EV isolation [109], although this method has predominantly been reported for isolation from cell culture media. Some of the recognised drawbacks of ultracentrifugation include protein aggregation and incomplete separation, as the size of pellets isolated is dependent upon the centrifugal force applied. Lower g forces risk contamination with apoptotic bodies [71], whereas higher centrifugal forces are considered to result in reduced yield due to EV damage [42]. Subsequent technical modifications of this include cushion ultracentrifugation and density gradient ultracentrifugation, which allow a more gentle separation of particles on the basis of density. Ultracentrifugation is also time-consuming, and in any event does not permit distinct isolation of those EVs specifically related to the disease of interest. That said, ultracentrifugation has still been used successfully for the isolation of melanoma derived EVs from human serum [58], as well as for the identification of potential subsequent melanoma-specific EV markers [25].

In light of the drawbacks associated with ultracentrifugation, other techniques are gaining in popularity [109]. Size-exclusion chromatography (SEC) is reported to preserve EV integrity [71]. It allows EVs larger than a defined size to be separated from other smaller particles on the basis of the time taken to pass through a column, and thus can result in higher yields [42]. Commercially available columns have been utilised to isolate melanoma-derived EVs from cell culture media, although their use has not yet been reported with serum or plasma from melanoma patients. Furthermore, SEC has been combined with other isolation methods, such as ultrafiltration and centrifugal filtration, resulting in reported higher yields of EVs in comparison to ultracentrifugation or precipitation methods [110]. Minimising the isolation of non-vesicular co-isolated components, for example, lipoproteins and albumin [13], remains a challenge, and sequential combinations of variations of UC and SEC [42,111] can improve this, and thus should be considered.

Precipitation agents have also increased in popularity [109], and commercially available agents such as ExoQuick have been utilised to isolate melanoma-derived EVs from both cell culture media [57] and patient serum [60]. However, while these agents isolate a high yield of EVs from serum, they also concentrate a higher proportion of “contaminating” smaller molecules, hypothesised to be protein aggregates or lipoproteins [42]. Finally, immunoaffinity-based methods of EV isolation have been increasingly employed [109], and have been used specifically to target melanoma derived-EVs in plasma, e.g., targeting the CSPG4 epitope on melanoma cells [50,55]. Importantly, this method may isolate EVs of different functional classes [110].

## 7. Challenges

Whilst the field of EVs is developing rapidly, there are many challenges and barriers to their introduction into clinical use. Inconsistent nomenclature and wide variations in both methods of isolation and reporting standards significantly hinder comparison between studies. These issues are being addressed by the EV community through the repeated revision of guidelines [13] and the introduction of an online database of experimental parameters EV-TRACK [112], which aims to encourage and facilitate systematic reporting on EV biology and methodology. Furthermore, although “exosomes” and other microvesicles such as “ectosomes” are considered to be functionally distinct, their current definitions somewhat overlap on the basis of their size, and may be co-isolated on the basis of existing techniques. Whilst some authors have made progress in this domain [19], further definition of markers to distinguish these subtypes is required, as well as establishing their functional relevance. However, these discrepancies will need to be addressed before the field can advance further.

In addition, the majority of studies performed on EVs in melanoma have been based upon homogenous cell line populations in vitro. Whilst cell lines have long been used to model melanoma molecular biology, they do not replicate the in vivo tumour microenvironment or immune landscape, and as such, they can be a poor representation of in vivo pathophysiology. Melanoma cell lines represent a valuable tumour model in terms of gene expression similarities, but also differ from their originating tumour at a transcriptional level [113]. Furthermore, EVs isolated from these cell lines represent those from a single cell clone and do not represent variations in tumour heterogeneity. This problem has been noted by those in the field, where melanoma-specific EV signatures developed in vitro are not replicable as reliably in vivo [35,114]. As such, further in vivo or ex vivo studies are required to determine the relevance of EVs in melanoma, and melanoma-specific EV signatures are required to address the heterogeneity of EVs in human plasma. This will also need to include the development of appropriate standards and biological controls, although some authors in clinical studies are already attempting to address this [47,88,115].

## 8. Conclusions

Melanoma prognostication and monitoring of treatment response remain a significant clinical challenge in both human and veterinary medicine, and despite advances in treatment, the disease still carries significant mortality. Melanoma-derived EVs have been identified in the circulation and have been demonstrated to play a significant role in tumorigenesis and disease progression. As a result, and due to the advantages they offer over other potential biomarker sources, EVs provide an attractive option for liquid biopsy in many species. In this regard, several clinical studies have attempted to define a melanoma-specific EV signature, and have shown their relevance in monitoring progression and response to treatment. As this field advances, in conjunction with standardisation of reporting and methodology, melanoma-derived EVs will likely play a key role in the clinical management and surveillance of all melanoma patients.

## Figures and Tables

**Table 1:** Potential melanoma-derived EV markers for diagnosis, as described in this review.

Protein Markers	Genetic Markers
HSP70 [25,32,52] Caveolin-1 [32,53] TRP-2 [25,54] Mel-CAM [25,52] Mart-1 [25,52,54] PMEL [32,49] CSPG4 [32,50,55] VLA-4 [25] MET [25]	miR-17 [56]
	miR-19a [56]
	miR-21 [56,57]
	miR-23 [57]
	miR-106b [58]
	miR-125b [57,59,60]
	miR-126 [56]
	miR-138 [57]
	miR-149 [56]
	miR-222 [57]
	miR-494 [57]
	miR-532-5p [58]
	miR-let7a-c [57]

**Table 2:** Summary of potential melanoma-derived EV protein markers as described in this review.

Protein Marker	Melanoma Cell Line	In Vivo	Function
HSP70	*SK-Mel-2* [32], *-5* [32], *-28* [25,32,52], *-202* [25], *-265* [25], *-35* [25]; *B16-F10* [25]; *LOX IMVI* [32]; *M14* [32]; *Malme-3M* [32]; *MeWo* [52]; *MDA-MB-435* [32]; *UACC-62* [32], *-257* [32]	Human plasma [25]	Detection [25]
Caveolin-1	*LOX IMVI* [32]; *M14* [32]; *Malme-3M* [32]; *Me501* [53]; *MDA-MB-435* [32]; *SK-Mel-2* [32], *-5* [32], *-28* [32]; *UACC-62* [32], *-257* [32]	Human plasma [53]	Detection [53]
Mel-CAM	*SK-Mel-28* [52]; *MeWo* [52]		Detection [25]
Mart-1	*SK-Mel-28* [52]; *MeWo* [52]; *RMS* [54]		Detection [25]
TRP-1	*RMS* [54]		Detection [25]
TRP-2	*B16-F10* [25]; *SK-Mel-28* [25], *-202* [25], *-265* [25], *-35* [25]; *RMS* [54]	Human plasma [25]	Detection [25], monitor progression [25]
PMEL	*LOX IMVI* [32]; *M14* [32]; *Malme-3M* [32]; *MDA-MB-435* [32]; *SK-Mel-2* [32], *-5* [32], *-28* [32]; *UACC-62* [32], *-257* [32]	Malignant effusions [49]	Detection [49]
VLA-4	*B16-F10* [25]; *SK-Mel-28* [25], -*202* [25], *-265* [25], *-35* [25]	Human plasma [25]	Detection [25]
CSPG4	*LOX IMVI* [32]; *M14* [32]; *Malme-3M* [32]; *MDA-MB-435* [32]; *SK-Mel-2* [32], *-5* [32], *-28* [32]; *UACC-62* [32], *-257* [32]	Plasma [50,55]	Detection [50,55]
MET	*B16-F10* [25]	Human plasma [25]	Detection [25], monitor progression [25]
MIA	*Malme-3M* [32]; *SK-Mel-2* [32], *-5* [32], *-28* [32]; *UACC-62* [32], *-257* [32]	Human plasma [77]	Monitoring progression [77]
S100B	*LOX IMVI* [32]; *Malme-3M* [32]; *MDA-MB-435* [32]; *SK-Mel-2* [32], -*5* [32], *-28* [32]; *UACC-257* [32]	Human plasma [77]	Monitoring progression [77]
PD-L1	*SK-Mel-2* [51]; *B16-F10* [51]	Human plasma [51,78]	Detection [78], monitoring progression [51]

**Table 3:** Summary of studies describing potential melanoma-derived EV genomic markers.

	Melanoma Cell Line	In Vivo	Genomic Markers Identified
Xiao et al. [57]	*A375*, *SK-Mel-28*		TOP1 mRNA, miR-21, miR-23, miR-125b, miR-138, miR-222, miR-494, miR-let7a/c
Gerloff et al. [59]	*WM9*, *WM35*, *WM902B*		miR-24-3p, miR-99b-5p, miR-100-5p, miR-125b-5p, miR-221-3p
Pfeffer et al. [56]		Plasma	miR-17, miR-19a, miR-21, miR-126, miR-149
Xiao et al. [85]	*A375*, *SK-Mel-28*	Serum	miR-191, miR-let-7a
Li et al. [86]	*WM35*, *A375*, *WM451*	Serum	miR-195-star, miR-494, miR-665
Tengda et al. [58]		Serum	miR-106b, miR-532-5p
